# Evaluations of the performances of PET and MRI in a simultaneous PET/MRI instrument for pre-clinical imaging

**DOI:** 10.1186/s40658-022-00483-x

**Published:** 2022-10-08

**Authors:** Alyssa C. Pollard, Jorge de la Cerda, F. William Schuler, Charles V. Kingsley, Seth T. Gammon, Mark D. Pagel

**Affiliations:** 1 Department of Chemistry, Rice University, Houston, TX USA; 2 Department of Cancer Systems Imaging, MD Anderson Cancer Center, Houston, TX USA; 3 Department of Imaging Physics, MD Anderson Cancer Center, Houston, TX USA

**Keywords:** PET/MRI, Image quality, Fluorine-18, Gallium-68, Quantification, Positron range

## Abstract

**Background:**

PET/MRI is an attractive imaging modality due to the complementary nature of MRI and PET. Obtaining high quality small animal PET/MRI results is key for the translation of novel PET/MRI agents and techniques to the radiology clinic. To obtain high quality imaging results, a hybrid PET/MRI system requires additional considerations beyond the standard issues with separate PET and MRI systems. In particular, researchers must understand how their PET system affects the MR acquisitions and vice versa. Depending on the application, some of these effects may substantially influence image quality. Therefore, the goal of this report is to provide guidance, recommendations, and practical experiments for implementing and using a small animal PET/MRI instrument.

**Results:**

Various PET and MR image quality parameters were tested with their respective modality alone and in the presence of both systems to determine how the combination of PET/MRI affects image quality. Corrections and calibrations were developed for many of these effects. While not all image characteristics were affected, some characteristics such as PET quantification, PET SNR, PET spatial resolution, PET partial volume effects, and MRI SNR were altered by the presence of both systems.

**Conclusions:**

A full exploration of a new PET/MRI system before performing small animal PET/MRI studies is beneficial and necessary to ensure that the new instrument can produce highly accurate and precise PET/MR images.

**Supplementary Information:**

The online version contains supplementary material available at 10.1186/s40658-022-00483-x.

## Background

Positron emission tomography (PET) and magnetic resonance imaging (MRI) are two crucial imaging modalities in both clinical and small animal studies for the identification and characterization of disease states. While PET and MRI can be performed sequentially, this imaging workflow can lead to difficulties in registering the PET and MR images. Sequential PET/MR imaging requires a long sum of scan times for a patient or small animal model and necessitates coordinating the schedules of two imaging instruments. Finally, interpreting results when a lengthy gap occurs between the separate PET and MRI scan sessions can be difficult, due to the potential change in morphology or physiology between the sessions.

To improve PET and MRI, efforts have been made to combine the two modalities into a single hybrid system for simultaneous PET/MRI [1]. The first PET/MR images were obtained in 1997, and the results were eventually translated into small animal studies [2]. PET/MRI technology expanded into human studies in 2008 when images of the human brain were acquired using a PET insert within a clinical 3 T MR scanner [3]. Finally, in 2010, the first clinical integrated whole-body PET/MRI scanner was commercially developed [4, 5]. Additional technical advancements such as improved PET detectors using silicon photomultipliers [6–8] and MR-based attenuation correction methods [9–12] have made PET/MRI more routine in the radiology clinic. Dedicated PET/MRI systems for small animal imaging have also been developed. Recently, PET inserts have been designed that can be placed within stand-alone small animal MRI magnets, which makes small animal PET/MRI more accessible [13–18].

With these hybrid imaging systems, issues may arise where one system may affect the performance of the other system due to the electronics or the presence of the magnetic field. For this reason, care must be taken to understand these effects so that they can be avoided or corrected. This report offers practical considerations and experiments that can be easily performed to understand the consequences of the combination of these imaging modalities. These considerations are not intended to be system-specific and should not be a replacement for NEMA characterization of a PET scanner or as official standards for MRI [19]. However, these considerations may eventually lead to these standards for PET/MRI. Finally, our report demonstrates advantages and disadvantages of simultaneous PET/MRI by providing insights on image quality parameters that are altered by the combination of the two systems.

## Methods

Details about PET reconstructions, MRI acquisition parameters, and MRI B_1_ and B_0_ homogeneity analysis are available in the Additional file 1.

### PET/MRI system description

All scans were performed on a Bruker 7 T MRI scanner (Bruker Biospin, Billerica, MA) with a Cubresa NuPET™ insert (Cubresa, Inc., Winnipeg, MB). Two 35 mm ^1^H volume transceiver coils were used to ensure that the PET/MRI evaluations were not dependent on a single MRI coil (Bruker Biospin, Billerica, MA and PulseTeq, Chobham, UK). PET/MR image registration and analysis were performed using VivoQuant v3.5 (Invicro, LLC, Boston, MA).

### PET quantification

A 3 mL tube was filled with either fluorine-18, gallium-68, copper-64, zirconium-89, or yttrium-86 and placed inside a 15 mL conical tube filled with 2% agarose. The starting activity was determined using a CRC-15R dose calibrator (Capintec, Inc., Florham Park, NJ). This tube was placed at axial center of the PET insert, and a 12 h scan was recorded. For long-lived nuclides, multiple 12 h scans were recorded. Results were divided into different activity levels (low: less than 13 MBq (350 µCi); medium: 13–23 MBq (351–620 µCi); high: greater than 23 MBq (621 µCi)). A Quantification Calibration Factor (QCF) was determined for each nuclide and activity level in the PET insert outside the MRI magnet. This experiment and analysis were repeated with the PET insert inside the MRI magnet using the Bruker 35 mm coil. QCFs were determined for certain nuclides without continuous MRI acquisitions or with a continuous RARE MRI or FISP MRI acquisition.

To test reproducibility, phantoms with 0.37–2.59 MBq (10–70 µCi) of fluorine-18 were each placed in axial center of the PET insert inside the MRI magnet. Each tube was scanned for 30 min along with a continuous RARE MRI acquisition, and the activity from the PET image was compared to activity measured by the dose calibrator. This experiment was repeated using phantoms with 0.37–1.67 MBq (10–45 µCi) of gallium-68. A Bland–Altman analysis [20] was performed to calculate the bias and 95% limits of agreement of the activity measurements.

### PET linear range

A Bland–Altman analysis [20] was performed using the QCF graphs for fluorine-18 and gallium-68 outside and inside the MRI magnet to determine how the linear range changes in the presence of the MRI magnet. The bias and 95% limits of agreement were calculated, and values within the 95% limits of agreement were considered within the linear range for that radionuclide.

### PET signal-to-noise (SNR)

The fluorine-18 datasets from the QCF analysis were used to calculate SNR for each 30 min time point. SNR was calculated by subtracting the signal from the noise from the true signal and then dividing by the standard deviation of the noise. This was performed for data with the PET insert outside the MRI magnet, inside the MRI magnet with no MRI acquisition, and inside the MRI magnet with a RARE MRI acquisition and a FISP MRI acquisition.

### PET Spatial resolution

A Derenzo phantom (Phantech Medical, Madison, WI) with node sizes of 1.0, 1.1, 1.2, 1.3, 1.4, and 1.5 mm was filled with ~ 3.7 MBq (100 µCi) of fluorine-18 and placed at axial center in the PET insert outside the MRI magnet. A 15 min PET scan was recorded. The phantom and insert were then moved into the MRI magnet with the Bruker 35 mm coil. A 15 min simultaneous PET/MRI scan was recorded using a continuous RARE MRI acquisition. This experiment was repeated using the Derenzo phantom filled with 3.7 MBq (100 µCi) of gallium-68.

### PET partial volume effects

A partial volume correction (PVC) phantom (Phantech Medical, Madison, WI) with 5.55 MBq (150 µCi) of fluorine-18 was placed inside the PET insert. Two 15 min PET scans were recorded with the phantom and insert inside the MRI magnet using the Bruker 35 mm MRI coil and outside the MRI magnet. Using the phantom’s reference region (which assumes 100% activity recovery), recovery coefficients were calculated for spheres of different diameters. This experiment was repeated using 9.25 MBq (250 µCi) of gallium-68.

### PET respiratory gating

The Derenzo phantom with ~ 3.7 MBq (100 µCi) of fluorine-18 was attached to a lever and placed in the axial center of the PET insert outside the MRI magnet. The phantom on the lever was manually moved up and down about 2–3 cm inside the PET insert continuously throughout a 15 min PET scan. A pneumatic pad was also placed inside the insert to detect the motion of the phantom (SA Instruments, Stony Brook, NY), which was relayed to the Cubresa software. PET image reconstruction could then be performed with respiratory gating by neglecting detected counts during periods of motion, or without respiratory gating by using all detected counts to reconstruct the image.

The phantom was also scanned with the PET insert inside the MRI magnet using the Bruker 35 mm MRI coil, moving up and down about 3–4 mm during a 15 min simultaneous PET/MRI scan using a continuous RARE MRI acquisition. PET image reconstruction was performed with and without respiratory gating. The experiment was repeated with ~ 3.7 MBq (100 µCi) of gallium-68.

### MRI signal-to-noise, linearity

A 15 mL conical tube filled with 20 mM CuSO_4_ was used in the following 6 scenarios: both 35 mm MR coils were tested without the PET insert, with the PET insert turned off, and with the PET insert turned on.

A MultiSlice, MultiEcho (MSME) spin-echo acquisition was optimized to test sensitivity and linearity. The average signal-to-noise of the tube in the axial image was plotted vs. slice position. The width and height of the tube was also measured in each axial image, and the diameter of the tube was plotted in each dimension vs. axial slice position to determine linearity. These tests were repeated for all 6 coil/insert scenarios. The tube of 20 mM CuSO_4_ was moved in the axial direction, and these scans were repeated to ensure the entire field of view (FOV) of both MR coils were included in the tests.

### MR representative images

Single-slice MSME, RARE, FLASH, MGE, True-FISP, FID-FISP, EPI, and UTE images were acquired in axial, coronal, and sagittal orientations. This set of images was repeated for all 6 coil/insert scenarios.

### MRI B_1_ and B_0_ homogeneity

A MSME image set was acquired with the same parameters used for the linearity tests. However, 45° and 90° excitation angles were used. The true excitation angle was then calculated along the axis of the sample. A B_0_ map was also created, which was plotted vs. axial position. These tests were repeated for all 6 coil/insert scenarios.

## Results

### PET quantification

**Fig. 1:**
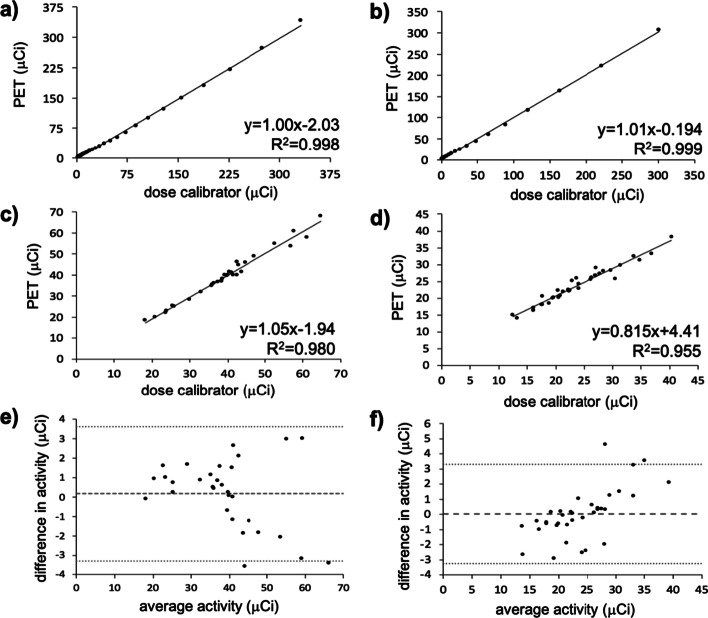
*PET radioactivity quantification in the presence of the MRI instrument.* Phantoms with (**a**) 12.58 MBq (340 µCi) of fluorine-18 and (**b**) 11.1 MBq (300 µCi) of gallium-68, were each placed in the axial center of the PET insert. The insert was loaded into the MRI instrument, and a simultaneous PET/MRI scan was recorded every 30 min over a 12 h time period. Each data point represents the amount of activity extrapolated from an initial measurement with a dose calibrator or measured from the PET image for each 30 min time point. The black line represents a linear fit of the data. Phantoms with (**c**) 0.37–2.59 MBq (10–70 µCi) of fluorine-18 or (**d**) 0.37–1.67 MBq (10–45 µCi) of gallium-68 were placed in the axial center of the PET insert. The insert was loaded into the MRI instrument, and each tube was scanned for 30 min using simultaneous PET/MRI. The black line represents a linear fit of the data. (**e**) A Bland–Altman analysis was performed for the data in Fig. 1c. The bias was 0.173 (dashed line), which was not significantly different than 0 (*p* = 0.432). The 95% limits of agreement were ± 3.47 (dotted lines). Linear regression analysis produced a slope of − 0.054, which was significantly different than 0 (*p* = 0.024). (**f**) A Bland–Altman analysis was performed for the data in Fig. 1d. The bias was 0.030 (dashed line), which was not significantly different than 0 (*p* = 0.489). The 95% limits of agreement were ± 3.30 (dotted lines). Linear regression analysis produced a slope of 0.183, which was significantly different than 0 (*p* = 1.17 × 10^–5^)

QCFs were determined for fluorine-18, gallium-68, copper-64, zirconium-89, or yttrium-86 inside and outside the MRI magnet by scanning the nuclides while they decayed over 10 half-lives (Fig. 1, Additional file 1: S1, S2). We found that a different QCF and normalization calibration were needed for different levels of activity (results for low-level activity shown in Figs. 1, Additional file 1: S1, S2; data not shown for medium and high activity levels). Different QCFs were also necessary when the PET insert was inside versus outside the MRI magnet. For the short-lived radionuclides (fluorine-18 and gallium-68), the ratio for the inside-to-outside the MRI magnet QCF was the same. In addition, the same QCF was valid whether or not a MRI pulse sequence was being performed. Different nuclides required different QCFs. Notably, these nuclide-dependent QCFs could not simply be calculated using the branching ratio of the respective nuclide, which emphasizes the need for experimental tests rather than calculations based on theory. After applying these unique QCFs to the datasets, it was determined that the PET insert can measure activity with an error of less than 3% for all radionuclides.

To test reproducibility, tubes were filled with fluorine-18 and placed in the axial center of the PET insert inside the MRI magnet. Each tube was scanned for 30 min, and the activity inside the tube was compared to the activity measured by the dose calibrator (Fig. 1c). This procedure was repeated with gallium-68 (Fig. 1d), and good agreement was observed between the PET insert and dose calibrator for both nuclides. Finally, a Bland–Altman analysis [20] was performed of these datasets to calculate the bias and 95% limits of agreement (Fig. 1e, f). For both nuclides, the bias was found to not be significantly different from zero (*p* > 0.05). However, a linear regression analysis of the data points on the Bland–Altman plots produced a slope that was significantly different than zero (*p* < 0.05). These results suggest an activity level-dependent bias with the PET insert quantification.

### PET linear range

A Bland–Altman analysis [20] was performed using the data in Additional file 1: Fig. S1a to determine the linear range of the PET insert for fluorine-18 and gallium-68 inside and outside the MRI magnet (Additional file 1: Fig. S2). Data points within the 95% limits of agreement were considered to be within the linear range. The linear range was minimally affected by the presence of the magnet. For fluorine-18 and gallium-68, a linear range was achievable up to 9.25 MBq (250 µCi). For both fluorine-18 scenarios, the bias and slope of the Bland–Altman data points were significantly different from zero. For gallium-68, this was also true for the scan performed outside the MRI magnet, but both the bias and slope were not significantly different from zero for the scan performed inside the MRI magnet.

### PET signal-to-noise

**Fig. 2:**
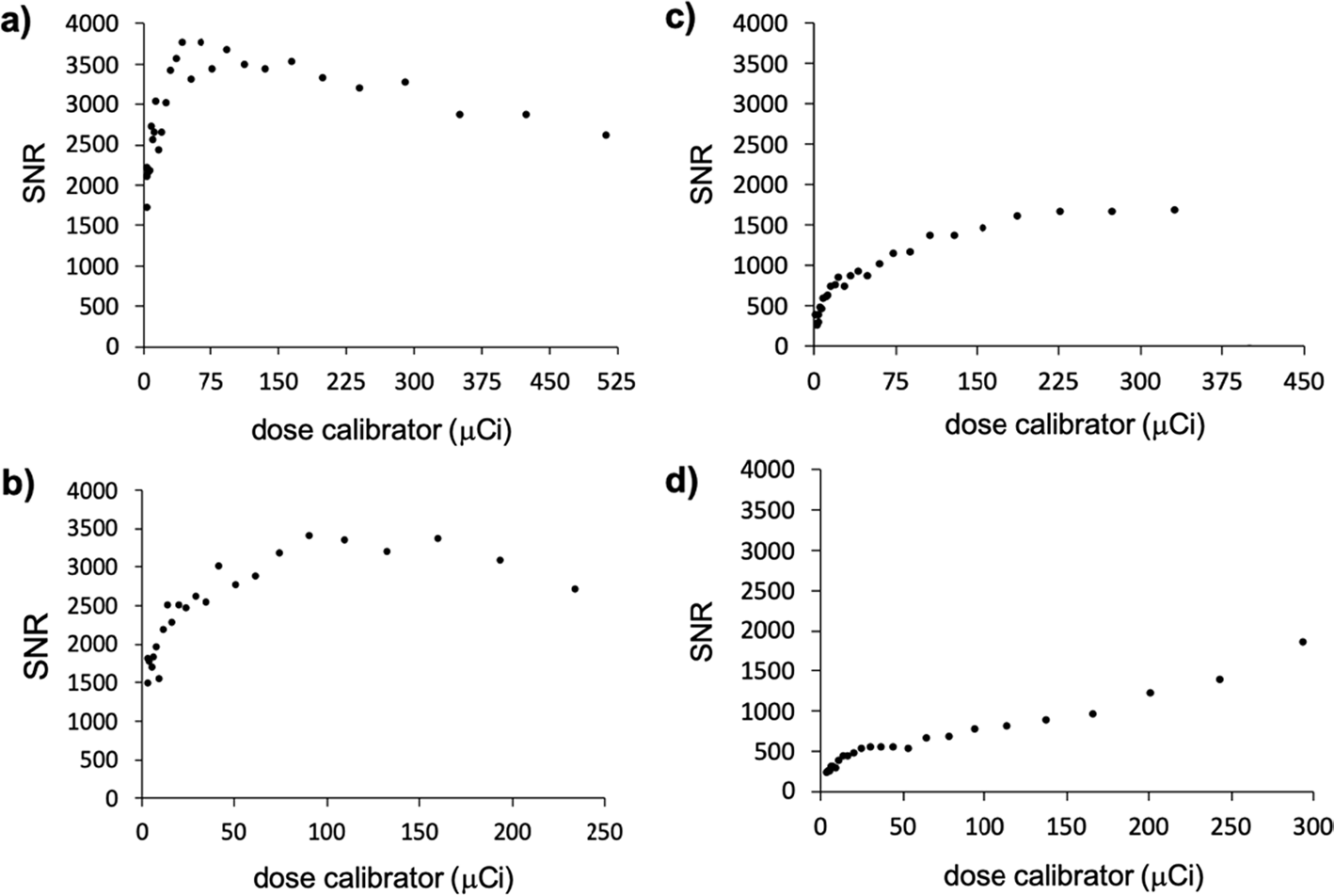
*PET SNR in the presence of the MRI instrument.* A phantom with fluorine-18 (**a**) at 19.43 MBq (525 µCi) was imaged using a PET insert outside the MRI magnet, (**b**) at 8.88 MBq (240 µCi) was imaged using a PET insert inside the MRI magnet without an active MRI acquisition, (**c**) at 12.58 MBq (340 µCi) was imaged using a PET insert inside the MRI magnet while a RARE MRI pulse sequence was run, and (**d**) at 11.1 MBq (300 µCi) was imaged using a PET insert inside the MRI magnet while a FISP MRI pulse sequence was run. Each data point represents a PET image that was recorded every 30 min over a 12 h time period. An ROI was drawn over the tube and also over a background region in each image to calculate SNR

Using the fluorine-18 data in Additional file 1: Fig. S1, an additional background ROI was drawn to calculate PET signal-to-noise. Equal levels of SNR could be achieved inside and outside the MRI magnet when there was no active MRI acquisition (Fig. 2a, b). However, when continuous RARE or FISP MR images were acquired, the SNR dropped to about half of the original value (Fig. 2c, d).

### PET spatial resolution

**Fig. 3:**
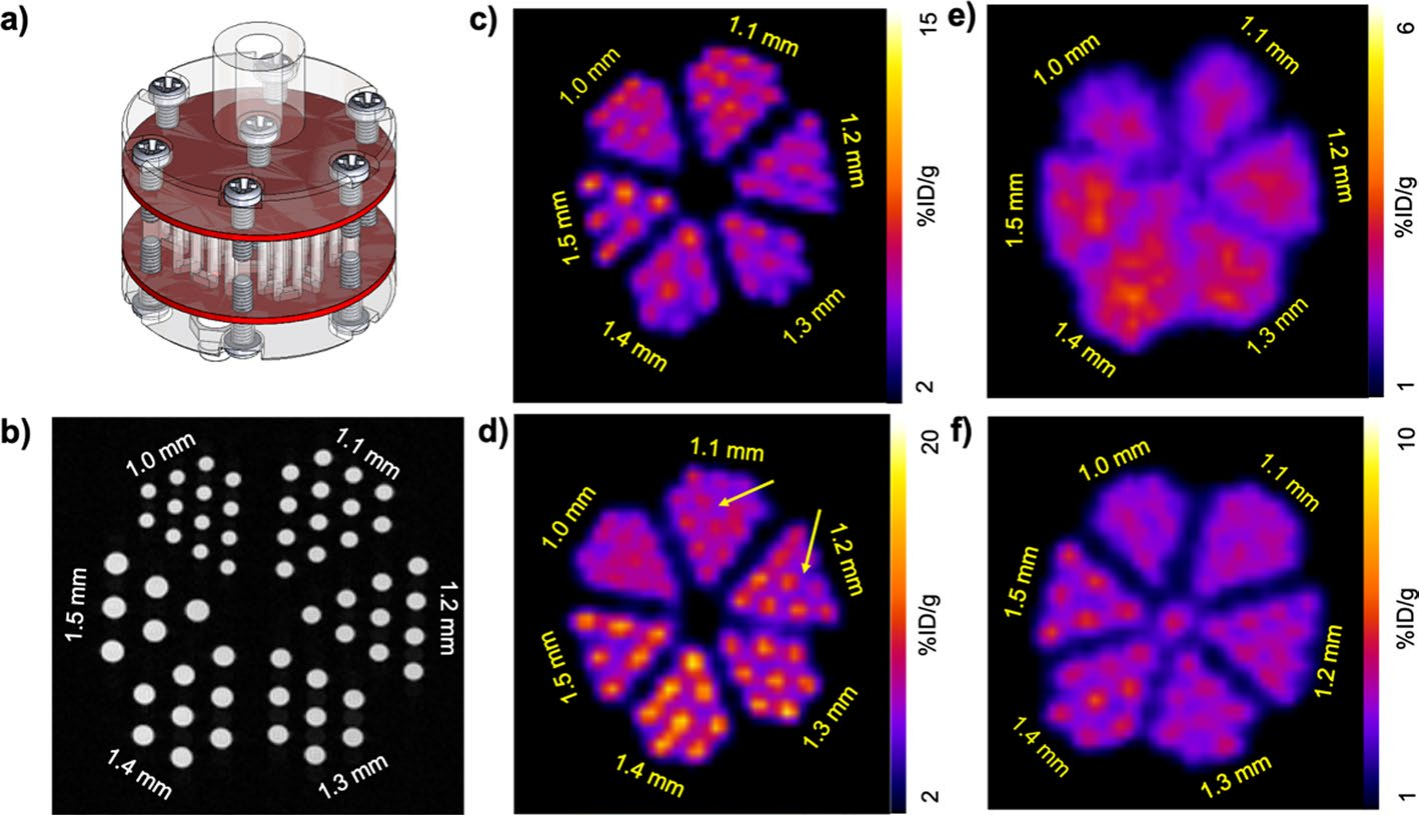
*PET spatial resolution in the presence of the MRI instrument.* **a** A schematic of the Derenzo phantom (image reproduced with permission from Phantech Medical). **b** MR image of the phantom. **c** The phantom with ~ 3.7 MBq (100 µCi) of fluorine-18 was placed at axial center in the PET insert. A spatial resolution of 1.0 mm was achievable. **d** The phantom of fluorine-18 and the PET insert were moved inside the MRI instrument. A spatial resolution of 1.0 mm was still achievable along with sharpening of the channels in some sections (yellow arrows). **e** The phantom with ~ 3.7 MBq (100 µCi) of gallium-68 was placed at axial center in the PET insert. The spatial resolution was greater than 1.5 mm. **f** The phantom with gallium-68 and the PET insert were moved inside the MRI instrument, and a spatial resolution of 1.2 mm was achievable

Spatial resolution of the PET and MRI imaging systems were each evaluated using a Derenzo phantom with channels ranging from 1.0 to 1.5 mm (Fig. 3a). Importantly, a quantitative measurement of PET image resolution requires a point source rather than a Derenzo phantom. Therefore, our results were used to evaluate the PET spatial resolution with the insert inside and outside the MRI magnet. For the MRI system in the presence of the PET insert, nodes with 1.0 mm diameter could easily be visually resolved, which is expected given the high spatial resolution of MRI (Fig. 3b). Using the phantom filled with fluorine-18, the nodes with 1.0 mm diameter could be visually resolved in the PET images when the insert was both inside and outside the MRI magnet (Figs. 3c, d). However, there was some sharpening of the images when the phantom was moved inside the MRI magnet (yellow arrows, Fig. 3d). For gallium-68, the PET spatial resolution drastically changed, from visually resolving nodes with 1.5 mm diameter outside the MRI magnet (Fig. 3e) to 1.2 mm inside the MRI magnet (Fig. 3f). These results suggest that radionuclide positron range can be influenced by the presence of the magnetic field of the MRI instrument.

### PET partial volume effects

**Fig. 4:**
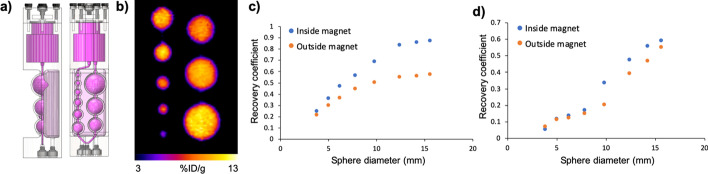
*PET partial volume effects in the presence of the MRI instrument.* **a** A schematic of the PVC phantom (image reproduced with permission from Phantech Medical). **b** A representative PET image of the phantom. **c** The phantom with 5.55 MBq (150 µCi) of fluorine-18 was placed in axial center of the PET insert inside and outside of the magnet. Recovery coefficients were higher when the PET scan was performed in the magnet. **d** The phantom with 9.25 MBq (250 µCi) of gallium-68 was placed in axial center of the PET insert inside and outside of the magnet. Recovery coefficients were higher when the PET scan was performed in the magnet

A PVC phantom was filled with fluorine-18 or gallium-68 and scanned with the PET insert inside and outside the MRI magnet. Similar to the resolution results (Sect. 3.4), lower recovery coefficients were observed outside the MRI magnet versus inside the MRI magnet (Fig. 4). Once again, this result suggests that the radionuclide positron range can be influenced by the presence of the MRI magnetic field.

### PET respiratory gating

**Fig. 5:**
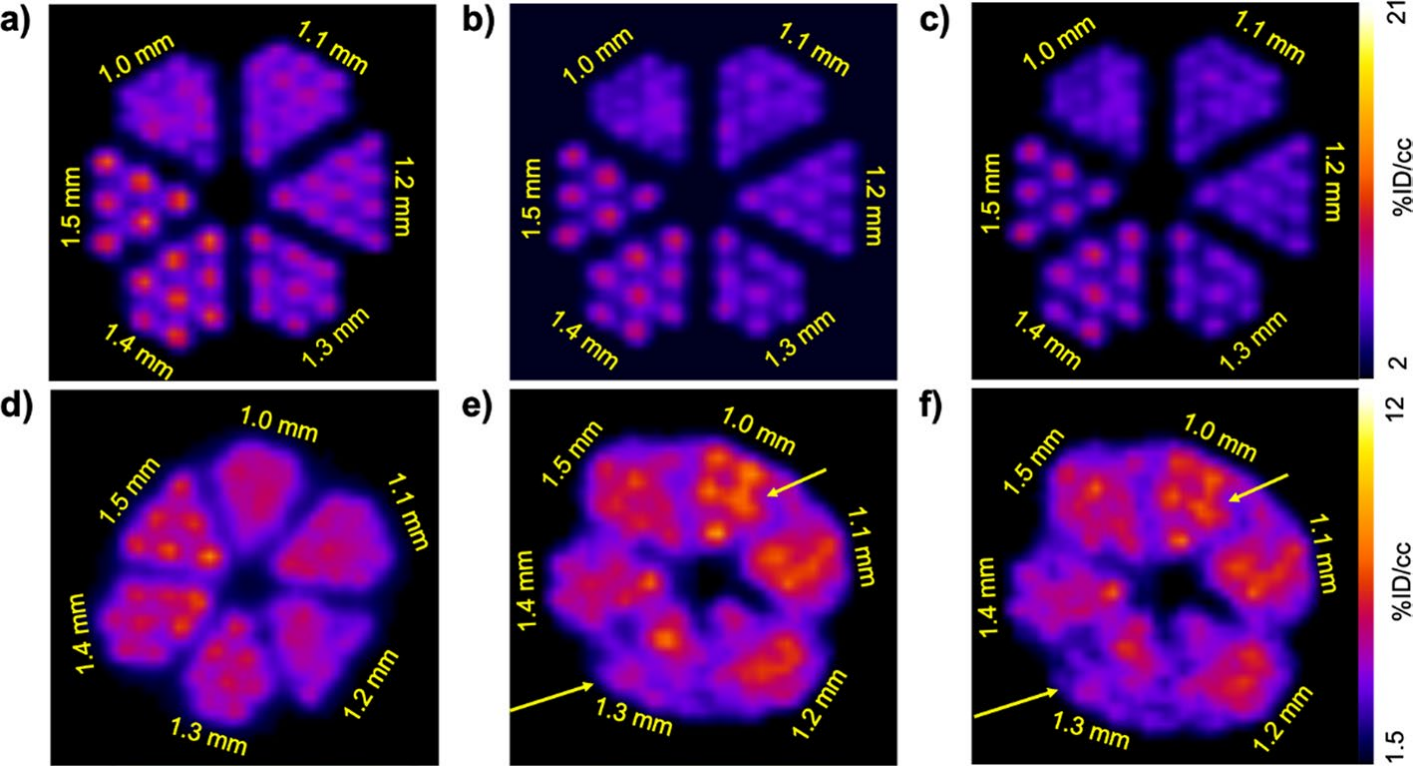
*PET respiratory gating in the presence of small motion artifacts.* **a** A Derenzo phantom (Phantech Medical) was filled with ~ 3.7 MBq (100 µCi) of fluorine-18, attached to a lever, and placed in the axial center of the PET insert inside the MRI scanner. A 15 min PET/MRI scan was performed with no phantom motion. **b** The phantom was artificially moved up and down about 3–4 mm inside the PET insert continuously throughout a 15 min PET/MRI scan to mimic the spatial scale of mouse respiratory motion. PET image reconstruction without respiratory gating produced very slight blurring. **c** PET image reconstruction with respiratory gating led to minimal PET image quality improvement. **d** The phantom with ~ 3.7 MBq (100 µCi) of gallium-68 was attached to a lever and placed in the axial center of the PET insert inside the MRI scanner. A 15 min PET/MR scan was performed with no phantom motion. **e** The phantom was artificially moved up and down about 3–4 mm inside the PET insert continuously throughout a 15 min PET/MR scan. PET image reconstruction without respiratory gating produced intense image blurring. **f** PET image reconstruction with respiratory gating led to sharpening of image resolution (yellow arrows)

Using the Derenzo phantom filled with fluorine-18, a large motion of 2–3 cm was simulated inside the PET insert but outside the MRI magnet continuously throughout a 15 min PET scan. The addition of respiratory gating in the PET reconstruction protocol prevented the appearance of a large motion artifact (Additional file 1: Fig. S3). The Derenzo phantom filled with fluorine-18 and then gallium-68 was also used with the PET insert inside the MRI magnet, and a smaller motion of 3–4 mm was artificially simulated to mimic the spatial scale of mouse respiratory motion. For fluorine-18, PET respiratory gating lead to minimal PET image quality improvement (Fig. 5b, c). However, for gallium-68, PET respiratory gating led to sharpening of the image spatial resolution (Fig. 5e, f).

### MRI signal-to-noise, linearity

**Fig. 6:**
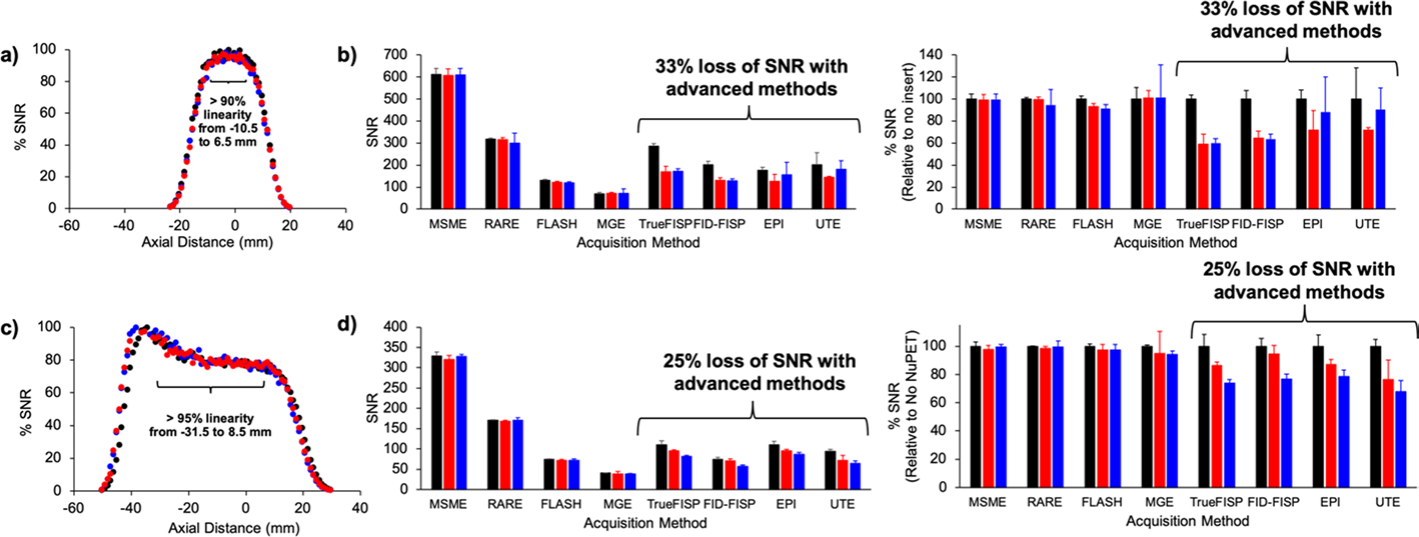
*MRI SNR in the presence of the PET insert.* **a** A 15 mL conical tube filled with a 20 mM CuSO_4_ solution was placed inside the MRI with the 35 mm Bruker MR coil and scanned with a MSME MRI acquisition without the PET insert (black), with the PET insert in the magnet and turned off (red), and with the PET insert in the magnet and turned on (blue). MR SNR and linearity were maintained throughout the axial FOV of the coil in all scenarios. **b** The PET insert did not affect SNR for MRI acquisition methods that are not gradient-intensive (MSME, RARE, FLASH, MGE). However, SNR decreased by 33% with gradient-intensive MRI acquisition methods (True-FISP, FID-FISP, EPI, uTE) when using the PET insert. **c** The same study was performed with the 35 mm PulseTeq MR coil. **d** The PET insert did not affect SNR for MRI acquisition methods that are not gradient-intensive (MSME, RARE, FLASH, MGE). However, SNR decreased by 25% with gradient-intensive MRI acquisition methods (True-FISP, FID-FISP, EPI, uTE) when using the PET insert. This loss of SNR was slightly more exaggerated with the insert turned on versus off when using the PulseTeq MR coil, but the opposite was true for the Bruker 35 mm MR coil

For MRI acquisition methods that were not gradient-intensive (MSME, RARE, FLASH, MGE), the presence of the PET insert did not affect SNR. However, there was a decrease in SNR with gradient-intensive MRI acquisition methods (True-FISP, FID-FISP, EPI, uTE) when using the PET insert (Fig. 6). For the Bruker 35 mm coil, the loss was 33% (Fig. 6b), and for the PulseTeq 35 mm coil, the loss was 25% (Fig. 6d). For these gradient-intensive sequences, the SNR was slightly lower with the insert on versus off for the PulseTeq coil, but the opposite was true for the Bruker coil. No change in MRI linearity was observed with or without PET insert for either MR coil (Additional file 1: Fig. S4). In addition, having the PET insert turned on or off did not affect linearity. The linearity only decreased outside of the axial FOV of the MR coil, which was about 4 cm for the PulseTeq 35 mm coil and about 2 cm for the Bruker 35 mm coil.

### MR representative images

The PET insert did not affect image quality as ascertained by a uniform signal amplitude throughout the tube of water and a lack of image artifacts in any orientation (Additional file 1: Fig. S5–S8). In addition, having the insert turned on or off did not lead to any changes in image quality. Any observed degradation in image quality was due to the MRI pulse sequence or markings on the sample tube and was independent of the PET insert.

### MRI B_1_ and B_0_ homogeneity

There was a sharp change in B_0_, the static magnetic field, at about + 15 mm along the axis of the PulseTeq coil (Additional file 1: Fig. S9), which has a ~ 5 cm axial field of view (Additional file 1: Fig. S4a). The standard shimming procedure used by our MRI instrument optimizes the B_0_ field within a 3 cm volume, so we attribute this sharp change to the edge of the shimming volume. The sharp change in B_0_ with the PulseTeq coil was exaggerated in the presence of the PET insert, indicating that the PET insert can change the B_0_ field, as expected. The PET insert may also affect B_0_ at this point for the Bruker 35 mm coil, but the small axial FOV of this coil (~ 3.5 cm as shown in Additional file 1: Fig. S4b) prevented measurements beyond + 10 mm. For comparison, the PET insert did not affect B_1_ power with either the PulseTeq 35 mm coil or the Bruker 35 mm coil (Additional file 1: Fig. S10).

## Discussion

PET radioactivity quantification is a key aspect to image analysis and can impact clinical decision making, which drives efforts to determine factors that affect quantification and ensure the accuracy and precision of PET inserts [21]. In our studies, we found that a lower QCF was required to accurately quantify activity inside the MRI magnet versus outside the MRI magnet. Interestingly, the ratio of the QCF for outside versus inside the MRI magnet was the same for short-lived radionuclides (fluorine-18 and gallium-68), indicating that this change in QCF is independent of the nuclide. In addition, this difference was not due to gradient switching because the same QCF was valid inside the MRI magnet whether or not an MRI acquisition was being performed. Therefore, we hypothesize that this increase in measured counts is due to the presence of the MRI magnet. More specifically, the MRI gradient cooling system surrounding the PET insert in the magnet may increase the efficiency of the general electronics in the system, leading to more recorded counts. Also, the presence of the magnetic field may possibly increase sensitivity, although the reason is unkown. A higher QCF and new normalization factors were also required for higher activity levels, which could be due to pile up of the PET detector crystals. We also observed that the linear range deteriorates at higher activity levels, which supports this hypothesis (data not shown). Finally, different nuclides required different QCFs, which is intuitive due to the different branching ratios of radionuclides. However, the branching ratio alone could not be used to calculate a valid QCF for different nuclides, which emphasizes the need for performing QCF experiments for each nuclide used in PET/MRI experiments.

We observed no change in PET SNR when the insert was in the magnet without acquiring a continuous MRI acquisition relative to PET imaging outside the MRI magnet. However, there was a two-fold decrease in SNR once a gradient-intensive MRI acquisition was started. It has been reported that PET background noise increases during MR image acquisition, which could lead to this decrease in PET SNR [22]. Further tests of count rates in the presence of gradient-intensive MRI acquisitions are required to further investigate how MRI affects PET SNR.

The distance that a positron travels before being annihilated by an electron is the positron range of that radionuclide. Due to the Lorentz force, magnetic fields cause emitted positrons to follow helical paths along the field lines. This decreases the positron range of radionuclides in the plane perpendicular to the magnetic field, and theoretically, leads to an improvement in PET spatial resolution in the transaxial direction [23–26]. We observed this improvement in PET transaxial spatial resolution for both fluorine-18 and gallium-68. This improvement was especially apparent for gallium-68, which has a higher positron range than fluorine-18. A shortening of the nuclide positron range was also apparent in our partial volume effects experiments, where we observed an increase in recovery coefficients when the phantom was inside the MRI magnet for both fluorine-18 and gallium-68. This result has been shown in other PET/MRI studies, where source size and magnetic field strength greatly influenced the spatial distribution of annihilation events [27]. Therefore, one advantage of PET/MRI is the improvement of PET spatial resolution and the decrease in partial volume effects. These improvements will be more prominent with small animal imaging, where a stronger magnetic field is used compared to clinical instruments [28].

The Lorentz force can also lead to potential shine-through artifacts on the PET image due to elongation of the positron range in the axial direction, and therefore, worsening spatial resolution in the axial direction [29]. However, this is mainly observed with very high energy radionuclides near air cavities, and we did not detect these artifacts in our phantom experiments possibly due to the nuclides we tested or because we placed our phantoms in a tube filled with agarose during all PET/MRI experiments. In addition, our Derenzo phantom was placed in the axial center of the PET/MRI system for all resolution experiments, so axial PET spatial resolution was not directly probed.

One additional advantage to our PET/MRI system is the ability to perform respiratory gating on both instruments simultaneously. This system was able to remove artifacts due to large motion over the course of a PET/MRI scan. While the high spatial resolution of MRI requires respiratory gating for most in vivo experiments, we did not find that PET respiratory gating was as beneficial for removing small motion artifacts. This is due to the lower spatial resolution of PET, which is often detector-limited and not affected by small motion. However, there were minor improvements observed when using radionuclides with a long positron range, such as gallium-68.

We observed that MRI SNR was not affected by the PET insert when using spin-echo sequences, but was affected when using gradient-intensive sequences, which agreed with a previous report [22]. Therefore, PET/MRI researchers should consider using spin-echo MRI acquisition methods during simultaneous PET/MRI. We did not observe degradation in MRI linearity or artifacts on MR images due to the presence of the PET, which we attribute to the sufficient RF shielding of the PET insert electronics. Other investigators have reported effects on MRI B_0_ field maps when the PET insert is in the magnet and turned on [22, 30]. Our results indicate that the presence of the PET insert can have some effect on the B_0_ field, so that shimming of the B_0_ field should be optimized with the PET insert in the magnet with the MRI coil, and preferably over the entire field of view of the MRI coil. In contrast, MRI B_1_ power was consistent in the presence of the PET insert for both MRI coils.

## Conclusions

In this report, we provide practical considerations when implementing a PET insert for small animal PET/MRI studies. Our results showed that many PET parameters such as quantification, SNR, resolution, and partial volume effects are altered due to the presence of the MRI instrument. However, MRI parameters are not affected by the presence of the PET insert with the exception of SNR. Based on these considerations, we encourage a full exploration of a new PET/MRI system before using it in small animal PET/MRI studies to ensure that the new instrument can produce highly accurate and precise PET/MRI data.

## Supplementary Information

**Additional file 1. **Supplementary materials are available that show additional details about the imaging materials and methods; PET QCFs; PET linear range; PET artifacts addressed by respiratory gating; MRI spatial linearity; MR image quality; and MRI B1 and B0 homogeneities.

## Data Availability

The datasets generated during and/or analyzed during the current study are available from the corresponding author on reasonable request.

## Abbreviations

EPI: Echo planar imaging
FISP: Fast imaging with steady-state free precession
FLASH: Fast low angle shot
FOV: Field of view
MGE: Multi gradient echo
MRI: Magnetic resonance imaging
MSME: Multi spin multi echo
NEMA: National electrical manufacturers association
PET: Positron emission tomography
QCR: Quantification calibration factor
RARE: Rapid acquisition with relaxation enhancement
ROI: Region of interest
SNR: Signal-to-noise ratio
UTE: Ultrashort time of echo
