# Supplementary material for: Evaluations of the performances of PET and MRI in a simultaneous PET/MRI instrument for pre-clinical imaging
Source: EJNMMI Phys. 2022 Oct 8;9:70. doi: 10.1186/s40658-022-00483-x (PMC9547760; doi:10.1186/s40658-022-00483-x)
Supplement: Supplementary file 1 — Additional file 1. Supplementary materials are available that show additional details about the imaging materials and methods; PET QCFs; PET linear range; PET artifacts addressed by respiratory gating; MRI spatial linearity; MR image quality; and MRI B1 and B0 homogeneities. [file 40658_2022_483_MOESM1_ESM.docx]

**SUPPLEMENTARY MATERIALS**

**Evaluations of the Performances of PET and MRI
in a simultaneous PET/MRI instrument for pre-clinical imaging**

R1.3

European Journal of Nuclear Medicine and Molecular Imaging

Alyssa C. Pollard^1,2^, Jorge de la Cerda^2^, F. William Schuler^2^, Charles V. Kingsley^3^, Seth T. Gammon^2^, Mark D Pagel^2,^*

^1^Department of Chemistry, Rice University, Houston, TX, USA

^2^Department of Cancer Systems Imaging, MD Anderson Cancer Center, Houston, TX, USA

^32^Department of Imaging Physics, MD Anderson Cancer Center, Houston, TX, USA

*Corresponding author: mdpagel@mdanderson.org

**Section 1. Materials and Methods**

Detailed Materials and Methods…………….………..……………………………………………………….. page S2

**Section 2. PET image parameters**

**Figure S1.** The effect of the MRI instrument on PET QCFs…………….………..………………………….. page S4

**Figure S2.** Bland-Altman plots allow for visualization of PET linear range…...…………………………. …. page S5

**Figure S3.** Artifacts caused by large motion can be prevented using PET respiratory gating ………… ……. page S5

**Section 3. MR image parameters**

**Figure S4.** MR linearity is not affected by the presence of a PET insert*.* ………………………….… ……… page S6

**Figure S5.** The effect of the PET insert on axial MR image quality using a 35 mm PulseTeq MR coil*.*…….. page S6

**Figure S6.** The effect of the PET insert on coronal MR image quality using a 35 mm PulseTeq MR coil. …. page S7

**Figure S7.** The effect of the PET insert on axial MR image quality using a 35 mm Bruker MR coil.…… …. page S7

**Figure S8.** The effect of the PET insert on coronal MR image quality using a 35 mm Bruker MR coil.… …. page S8

**Figure S9.** A PET insert can cause a significant change in B_0_ with long FOV MR coils. …………………... page S8

**Figure S10.** B_1_ power is not affected by the presence of a PET insert*.*…….………………………… ……... page S9

**S1. Materials and Methods**

Details about PET reconstructions, MRI acquisition parameters, and MRI B_1_ and B_0_ homogeneity analysis are available in this section.

*S1.1. PET/MRI System Description*

Corrections for random coincidences and radioactive decay were applied through the Cubresa software. PET reconstructions were performed through the Cubresa software using an Ordered Subset Maximum a Posteriori One Step Late (OSMAPOSL) algorithm with 8 iterations and 4 subsets, unless otherwise stated. MR reconstructions were performed using ParaVision 6.0.1 (Bruker Biospin, Billerica, MA).

*S1.2. PET Quantification*

The PET data was binned into 30 min time frames and reconstructed. A VOI was drawn around the entire tube of activity, and the sum of the activity in that VOI was calculated by the analysis software. This value was plotted for each 30 min time point versus the decay-calculated activity starting from the dose calibrator value. Using the slope of the data, a Quantification Calibration Factor (QCF) was determined for each nuclide and activity level in the PET insert outside the MRI magnet. This unique QCF was then applied to the datasets during a new reconstruction, the data was plotted, and new slopes were calculated to determine the PET insert’s ability to measure radioactivity. During the PET scans inside the MRI magnet with an active MRI acquisition, either a continuous RARE MRI acquisition (TE = 21.68 ms; TR = 2166.375 ms; RARE factor = 8; echo spacing = 5.420 ms; 1 average; 1275 repetitions; 25 slices; slice thickness = 2 mm; 38.4 x 38.4 mm FOV; 128 x 128 matrix; 300 x 300 μm resolution), or a continuous FISP MRI acquisition (TE = 1.655 ms; TR = 3.310 ms; flip angle = 15°; 30 averages; 3480 repetitions; 1 slice; slice thickness = 2 mm; 80 x 80 mm FOV; 128 x 128 matrix; 625 x 625 μm resolution) was used. For the reproducibility tests, a Bland-Altman analysis was performed using Microsoft Excel (Microsoft, Corp., Redmond, WA).

*S1.3. PET Linear Range*

A Bland-Altman analysis was performed using Microsoft Excel (Microsoft, Corp., Redmond, WA) using the QCF graphs for fluorine-18 and gallium-68 outside and inside the MRI magnet to visually determine how the linear range changes in the presence of the MRI magnet. The bias and 95% limits of agreement were calculated, and values within the 95% limits of agreement were considered within the linear range for that radionuclide.

*S1.4. PET Signal-to-Noise (SNR)*

Using the fluorine-18 datasets from the QCF analysis, an additional VOI was drawn with the same dimensions as the tube VOI in a region where the tube was not located to represent background noise. The VOI from Section S1.2 and the standard deviation of this new background VOI were used to calculate SNR for each 30 min time point.

*S1.5. PET Spatial Resolution*

The PET scan was recorded and reconstructed using a Point Spread Function-Ordered Subset Expectation Maximization (PSF-OSEM) algorithm with 8 iterations and 4 subsets to determine the spatial resolution of the insert alone. A continuous RARE MRI acquisition was used with the insert inside them magnet using the same parameters as listed in section S1.2.

*S1.6. PET Respiratory Gating*

A continuous RARE MRI acquisition was used with the same parameters as listed in Section S1.2. PET image reconstruction was performed with and without respiratory gating applied using a PSF-OSEM algorithm with 8 iterations and 4 subsets.

*S1.7. MRI Signal-to-Noise, Linearity*

A 15 mL conical tube filled with 20 mM CuSO_4_ was used in the following experiments. The T_1_ relaxation time of this sample was 75 ± 1 ms. Therefore, a repetition time (TR) of at least 400 ms was used for each scan to ensure TR > 5*T_1_.

A MultiSlice, MultiEcho (MSME) spin echo acquisition was optimized to test sensitivity and linearity (TR = 2000 ms; TE = 8.0 ms; 90 degree excitation angle; 128 x 128 matrix; FOV = 38.4 x 38.4 mm; in-plane resolution = 300 x 300 μm; slice thickness = 1 mm; 120 slices; interlaced slice acquisition; 4 dummy scans; 1 average). The average signal-to-noise of the tube in the axial image was plotted vs. slice position. The noise was determined from a 1x1 cm square in an area of the image that represented air, with no apparent artifacts, and with care to avoid the fiducial markers built into the coil. The standard deviation of the noise was used for the SNR calculation.

*S1.8. MR Representative Images*

Single-slice MSME images were acquired in axial and coronal orientations. The coronal images had a 38.4 x 153.6 mm FOV with a 128 x 512 matrix size, for an in-plane resolution of 300 x 300 μm. All images used a 400 ms TR. Otherwise, all other parameters were the same as the multislice MSME described above.

In addition to MSME, single-slice RARE, FLASH, MGE, True-FISP, FID-FISP, EPI and UTE images were acquired in the axial, coronal, and sagittal orientations, using the same dimensions as the single-slice MSME images. The RARE acquisition had a RARE factor = 16; TE = 6 ms; effective TE = 48 ms. The FLASH had a TR = 20 ms; TE = 2.68 ms, flip angle = 10 degrees. The MGE had TR = 50 ms; TE = 2.040 ms; 10° flip angle; 6 echos, 5 ms echo spacing. The True-FISP had TR = 3.31 ms; TE = 1.655 ms; 60° flip angle; “starter sequence” SSFP preparation with a 30° flip angle for Mz and 8 preparations for Mxy. The FID-FISP had the same parameters as the True-FISP but with an FID mode. The EPI had TR = 400 ms; TE = 15.44 ms (42.32 ms for sagittal and coronal orientations); 200,000 Hz bandwidth, in spin echo mode. The UTE had TR = 10 ms; TE = 1.629 ms; ECHO acquisition mode; 202 projections, 100,000 Hz bandwidth; 15° flip angle, 120 dummy scans. The UTE sequence must have a square matrix, so the sagittal and coronal images were acquired with a 512 x 512 matrix, 15.36 x 15.36 cm FOV, 300 x 300 μm resolution, which changed TR = 12.866 ms and TE = 3.549 ms, with 804 projections. These scans were repeated for all 6 coil/insert scenarios.

*S1.9. MRI B_1_ and B_0_ Homogeneity*

A MSME image set was acquired with the same parameters used for the linearity tests. However, 45° and 90° excitation angles were used. The true excitation angle was then calculated using the following formula: Y=(360*(acos(X(1)/(2*X(2)))))/(2*π), where: “acos” is an arccos function; X(1) is the signal amplitude for the image with the 90° excitation; and X(2) is the signal amplitude for the image with the 45° excitation. Notably, the value of π should be used with at least 6 decimal points for an accurate calculation due to the nature of an arccos function. The average excitation angle was calculated along the axis of the sample. This test was repeated for all 6 coil/insert scenarios. Care was taken to ensure that both MR image sets with 45° and 90° excitation angles were acquired with the same receiver gain value, so that the amplitudes of both image sets could be compared.

A B_0_ map was created using ParaVision 6.0.1 (Bruker Biospin, Billerica, MA), which was plotted vs. axial position. This method obtains 64 B_0_ maps with a 0.906 slice thickness. Other parameters were TR = 20 ms; TE = 1.95 ms; 1 average; 30° excitation angle; 2 echo images; 58 mm isotropic FOV with 64x64x64 matrix (0.906 μm resolution in each dimension). This test was repeated for all 6 coil/insert scenarios.

**S2. PET image parameters**

**
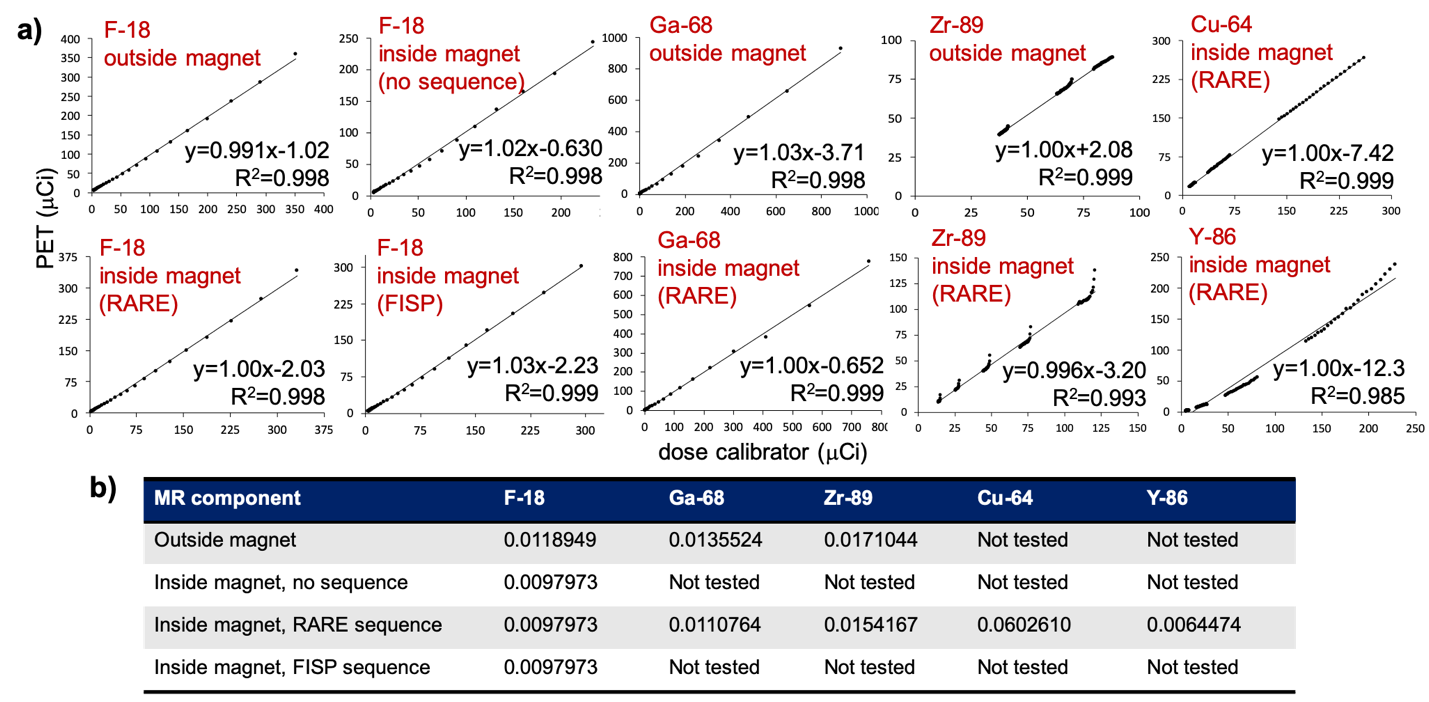
**

R1.4

**Fig. S1** *The effect of the MRI instrument on PET QCFs.* a) Tubes filled with a radionuclide were each placed in the axial center of the PET insert. For scans performed inside the MRI magnet, the insert was loaded into the magnet, the MRI coil was placed in the PET insert, and the tube was placed inside the MRI coil. For both PET only and simultaneous PET/MRI experiments, a PET scan was recorded every 30 min over a 12 h time period (for long-lived zirconium-89, copper-64, and yttrium-86 nuclides, this 12 h scan was performed for three days). The slope of each graph was used to create a unique QCF for each nuclide and scenario (graphs not shown). The QCF was then applied to the data during PET reconstruction, producing great agreement between the dose calibrator and PET insert. Each data point represents the amount of activity extrapolated from an initial measurement with a dose calibrator or measured from the PET image for each 30 min time point. The black line represents a linear fit of the data. b) Different QCFs were produced for nuclides at activity levels less than 13 MBq (350 µCi) and different PET insert scenarios. Different QCFs were necessary when the PET insert was inside versus outside the MRI magnet. However, the same QCF for studies inside the MRI magnet was valid whether or not an MRI acquisition was being run. Different nuclides also required different QCFs.


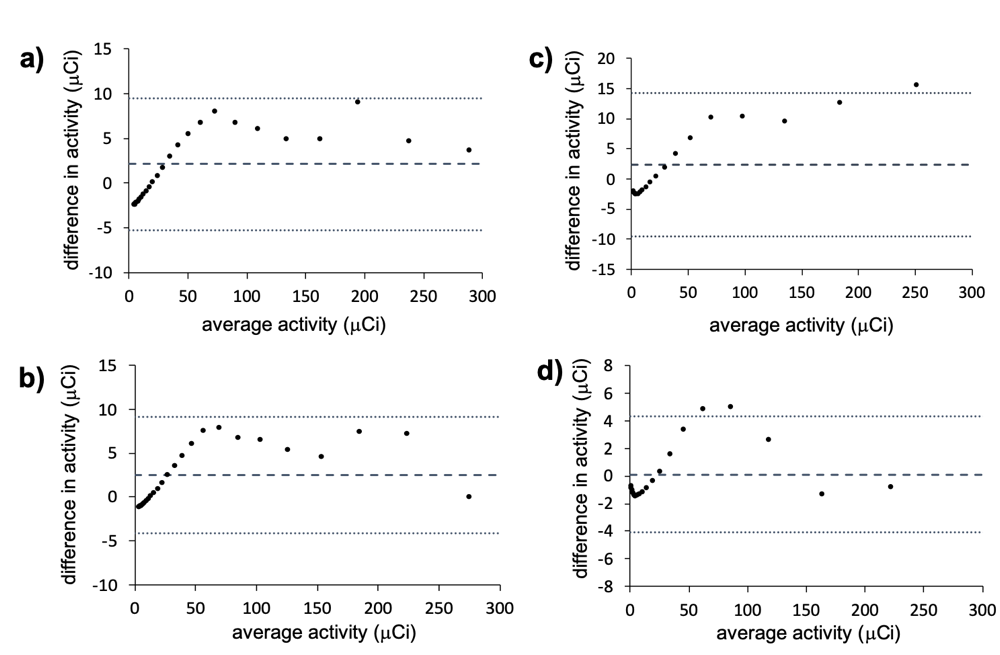


R1.4

**Fig. S2** *Bland-Altman plots of the PET linear range.* A Bland-Altman analysis was performed for some of the data in Figure S1 including a) fluorine-18 outside the MRI magnet, b) fluorine-18 inside the MRI magnet with a continuous RARE MRI acquisition, c) gallium-68 outside the MRI magnet, and d) gallium-68 inside the MRI magnet with a continuous RARE MRI acquisition. The dashed lines represent the bias, and the dotted lines represent the 95% limits of agreement. The PET linear range was not affected by the presence of MRI. For both fluorine-18 and gallium-68, a linear range was achieved up to 9.25 MBq (250 µCi).


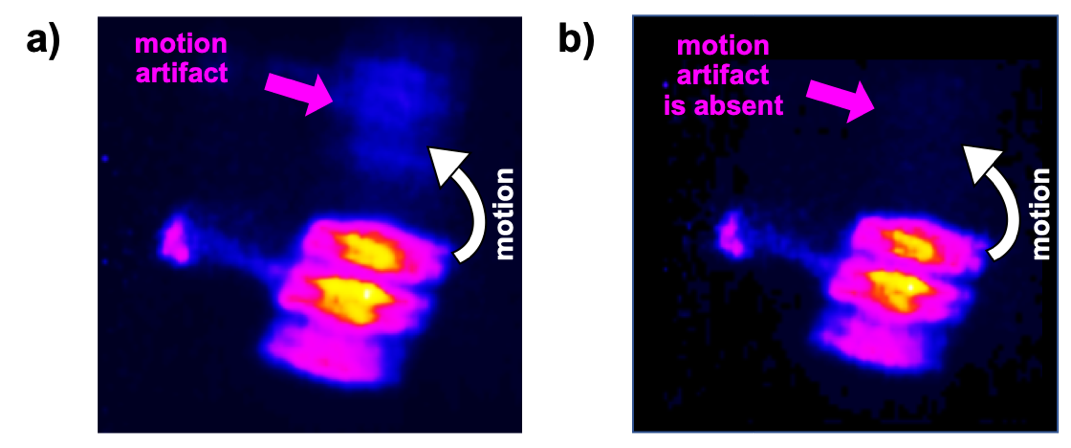


R1.4

**Fig. S3** *PET respiratory gating.* a) A Derenzo phantom (Phantech Medical) was filled with ~0.37 MBq (100 µCi) of fluorine-18, attached to a lever, and placed in the axial center of the PET insert (no MRI present). The phantom was manually moved up and down about 2-3 cm inside the PET insert continuously throughout a 15 min scan. PET image reconstruction without respiratory gating led to motion artifacts. b) PET image reconstruction with respiratory gating removed the motion artifact.

**S3. MR image parameters**

**
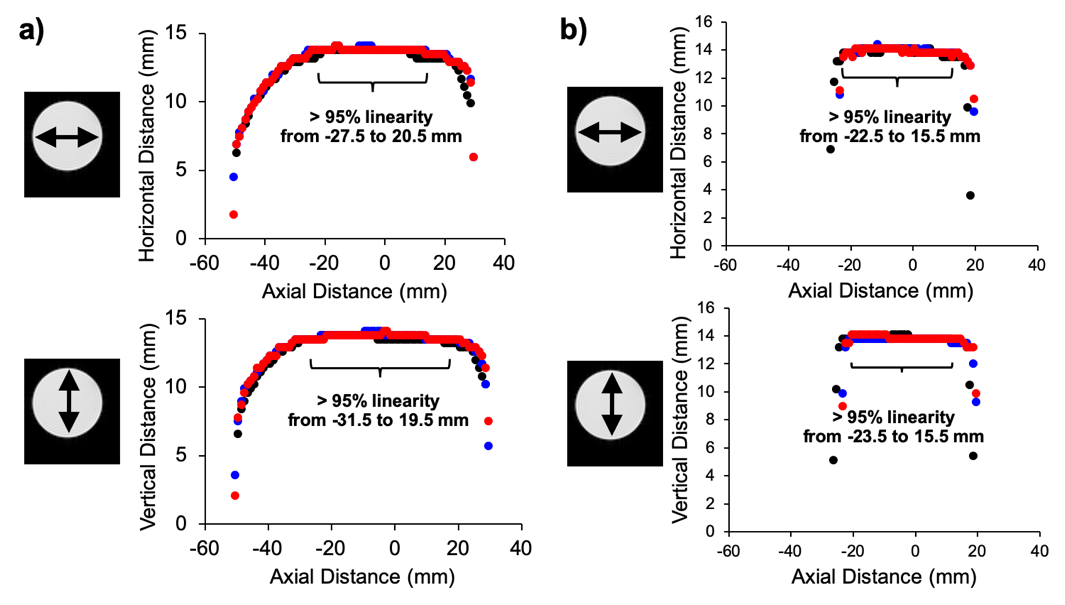
**

R1.4

**Fig. S4** *MR linearity in the presence of a PET insert.* A 15 mL conical tube filled with a 20 mM CuSO_4_ solution was placed at axial center of the MRI and scanned without the PET insert (black), with the PET insert turned off (red), or with the PET turned on (blue)*.* The width and height of the tube was recorded in each MR axial image, and the diameter of the tube was plotted in each dimension versus axial slice position to determine linearity. These experiments were performed using a) a PulseTeq 35 mm coil and b) a Bruker 35 mm coil. While the coils have different linear FOVs, no change in MR linearity was observed due the PET insert for either coil.


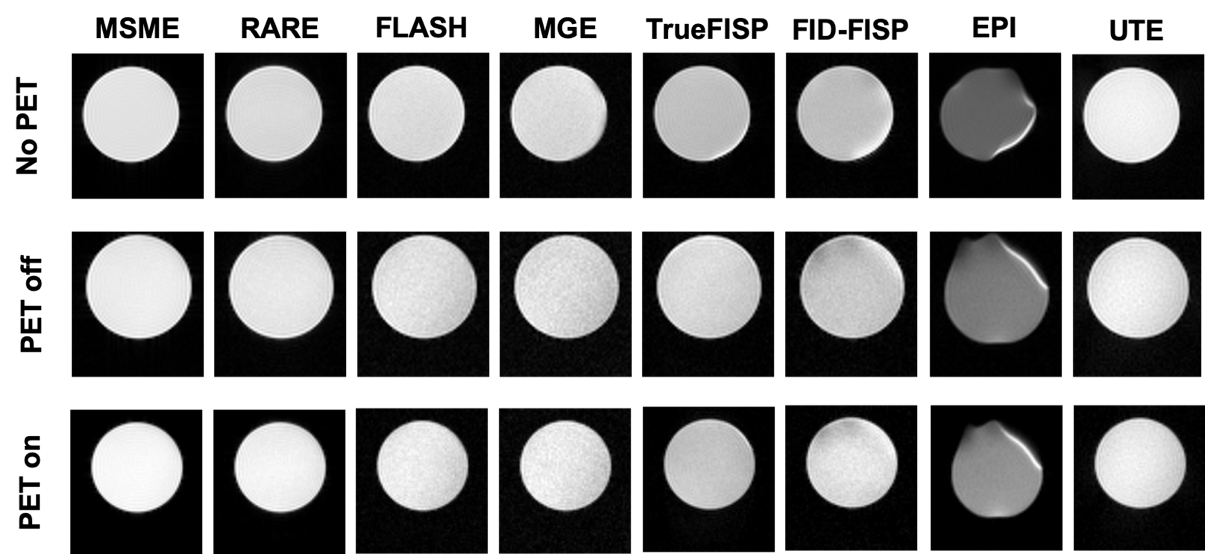


R1.4

**Fig. S5** *The effect of the PET insert on axial MR image quality using a 35 mm PulseTeq MR coil.* Representative axial MR images of a 15 mL conical tube filled with a 20 mM CuSO_4_ solution were obtained using various MR sequences (columns). For each sequence, an image was acquired without the PET insert in the magnet (top row), with the insert in the magnet but turned off (middle row), and with the insert in the magnet and turned on (bottom row). The PET insert did not affect axial MR image quality. Notably, the EPI image showed a distortion without the PET insert as well as with the PET insert, indicating that EPI images were distorted by the MRI instrument and not by the PET insert.

**
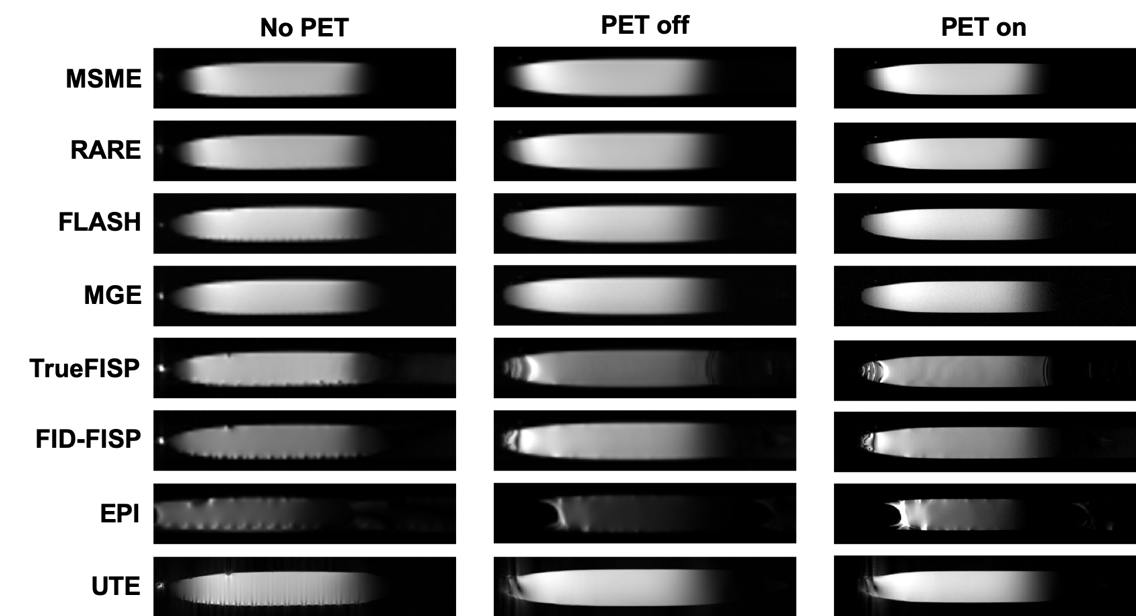
**

R1.4

**Fig. S6** *The effect of the PET insert on coronal MR image quality using a 35 mm PulseTeq MR coil.* Representative coronal MR images of a 15 mL conical tube filled with a 20 mM CuSO_4_ solution were obtained using various MR sequences (rows). For each sequence, an image was acquired without the PET insert in the magnet (left column), with the insert in the magnet but turned off (middle column), and with the insert in the magnet and turned on (right column). The PET insert did not affect coronal MR image quality. Notably, the EPI image showed a distortion without the PET insert as well as with the PET insert, indicating that EPI images were distorted by the MRI instrument and not by the PET insert. In addition, artifacts in the TrueFISP, FID-FISP, and UTE were caused by the ink in the graduation marks on the 15 mL conical tube, which caused B_0_ inhomogeneities and were not a product of the PET insert.

**
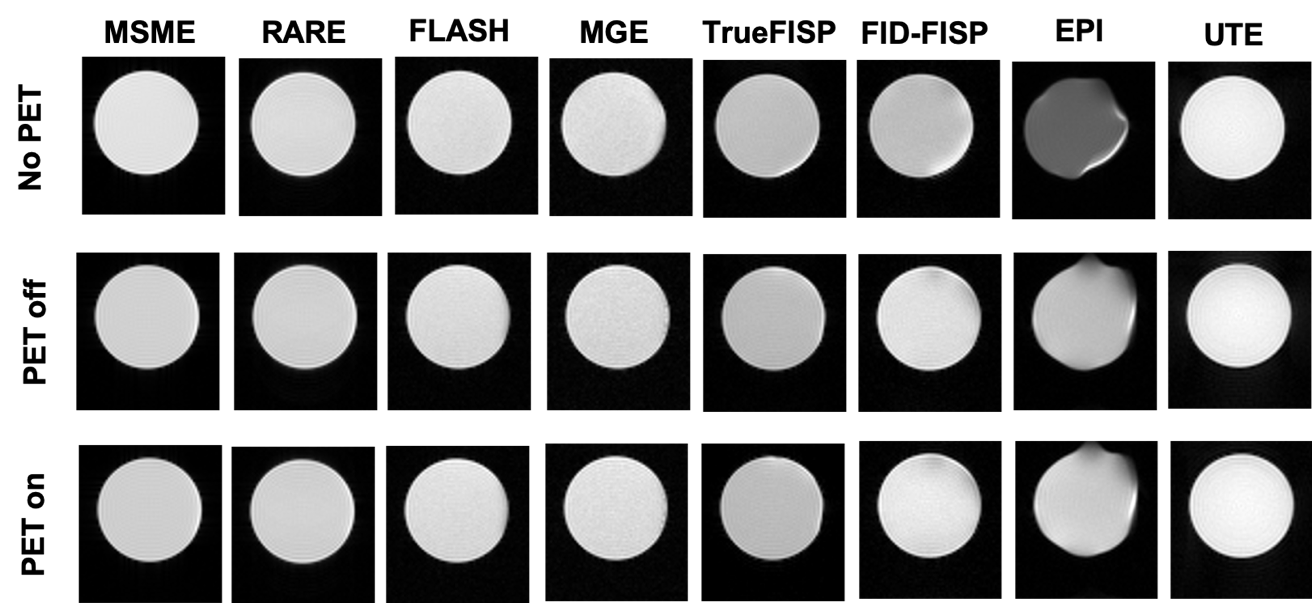
**

R1.4

**Fig. S7** *The effect of the PET insert on axial MR image quality using a 35 mm Bruker MR coil.* Representative axial MR images of a 15 mL conical tube filled with a 20 mM CuSO_4_ solution were obtained using various MR sequences (columns). For each sequence, an image was acquired without the PET insert in the magnet (top row), with the insert in the magnet but turned off (middle row), and with the insert in the magnet and turned on (bottom row). The PET insert did not affect axial MR image quality. Notably, the EPI image showed a distortion without the PET insert as well as with the PET insert, indicating that EPI images were distorted by the MRI instrument and not by the PET insert.

**
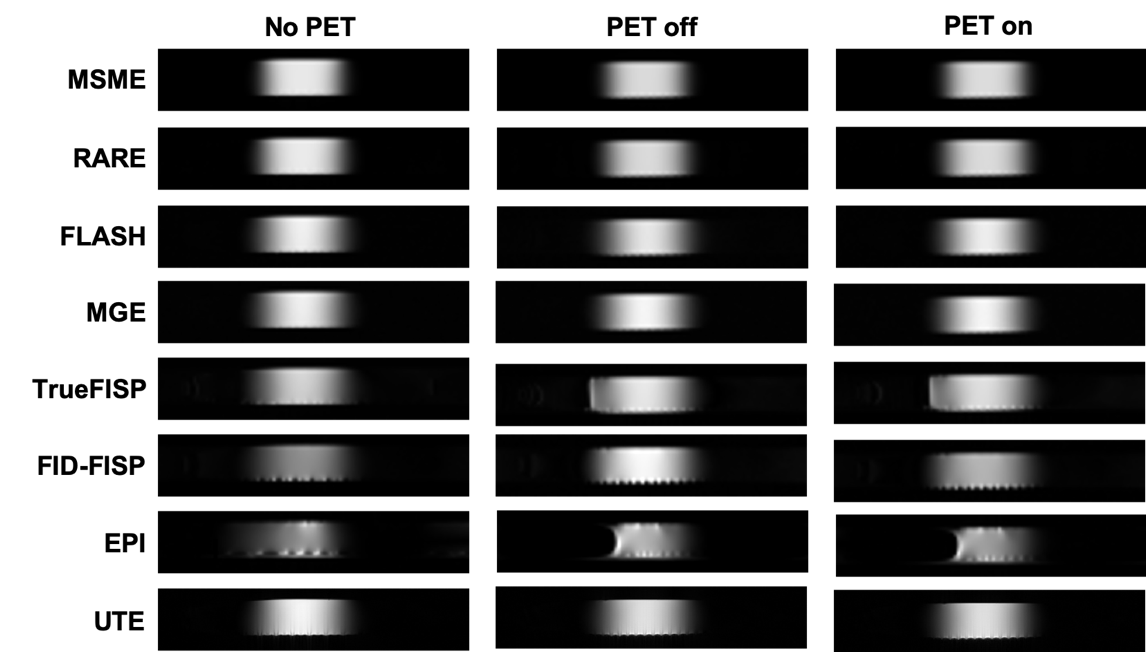
**

R1.4

**Fig. S8** *The effect of the PET insert on coronal MR image quality using a 35 mm Bruker MR coil.* Representative coronal MR images of a 15 mL conical tube filled with a 20 mM CuSO_4_ solution were obtained using various MR sequences (rows). For each sequence, an image was acquired without the PET insert in the magnet (left column), with the insert in the magnet but turned off (middle column), and with the insert in the magnet and turned on (right column). The PET insert did not affect coronal MR image quality. Notably, the EPI image showed a distortion without the PET insert as well as with the PET insert, indicating that EPI images were distorted by the MRI instrument and not by the PET insert. In addition, artifacts in the TrueFISP, FID-FISP, and UTE were caused by the ink in the graduation marks on the 15 mL conical tube, which caused B_0_ inhomogeneities and were not a product of the PET insert.

**
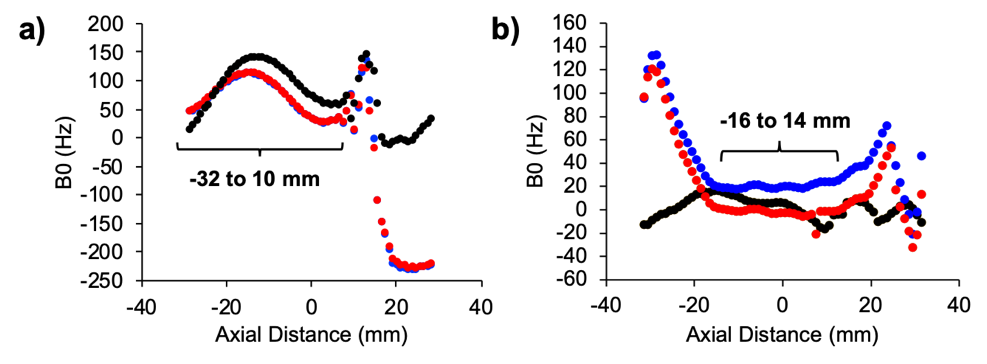
**

R1.4

**Fig. S9** *B_0_ in the presence of a PET insert.* A 15 mL conical tube filled with a 20 mM CuSO_4_ solution was placed at axial center of the MRI and scanned without the PET insert (black), with the PET insert turned off (red), or with the PET turned on (blue)*.* a) When using a PulseTeq 35 mm coil, a major jump in B_0_ was observed at about +15 mm along the axial direction, and this jump was exaggerated in the presence of the PET insert. b) No changes in B_0_ were observed when using the shorter FOV Bruker 35 mm coil. The PET insert may also affect B_0_ at the same point for the Bruker 35 mm coil, but the small axial FOV of this coil prevented measurements beyond +10 mm.

**
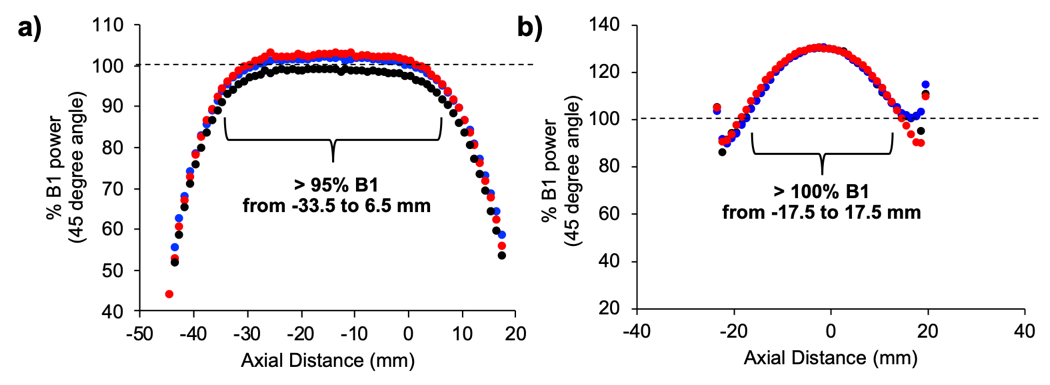
**

R1.4

**Fig. S10** *B_1_ power in the presence of a PET insert.* A 15 mL conical tube filled with a 20 mM CuSO_4_ solution was placed at axial center of the MRI and scanned without the PET insert (black), with the PET insert turned off (red), or with the PET turned on (blue). This was performed using a) a Pulse Teq 35 mm coil and b) a Bruker 35 mm coil. While the coils have different linear FOVs, no change in B_1_ power was observed due the PET insert for either coil.
